# Phytochemical Profile and Bioactivity of Bound Polyphenols Released from *Rosa roxburghii* Fruit Pomace Dietary Fiber by Solid-State Fermentation with *Aspergillus niger*

**DOI:** 10.3390/molecules29081689

**Published:** 2024-04-09

**Authors:** Qing Chen, Juan Su, Yue Zhang, Chao Li, Siming Zhu

**Affiliations:** 1 School of Food Science and Engineering, South China University of Technology, Guangzhou 510640, China; chenqing28532@163.com (Q.C.); benbenhu5@163.com (J.S.); zy17733135750@163.com (Y.Z.); 2 School of Food and Health, Guangzhou City Polytechnic, Guangzhou 510405, China; 3 Guangdong Province Key Laboratory for Green Processing of Natural Products and Product Safety, Guangzhou 510640, China

**Keywords:** *Rosa roxburghii* fruit pomace, agricultural byproduct, bound polyphenols, fermentation

## Abstract

This study aimed to investigate the phytochemical profile, bioactivity, and release mechanism of bound polyphenols (BPs) released from *Rosa roxburghii* fruit pomace insoluble dietary fiber (RPDF) through solid-state fermentation (SSF) with *Aspergillus niger*. The results indicated that the amount of BPs released from RPDF through SSF was 17.22 mg GAE/g DW, which was significantly higher than that achieved through alkaline hydrolysis extraction (5.33 mg GAE/g DW). The BPs released through SSF exhibited superior antioxidant and α-glucosidase inhibitory activities compared to that released through alkaline hydrolysis. Chemical composition analysis revealed that SSF released several main compounds, including ellagic acid, epigallocatechin, *p*-hydroxybenzoic acid, quercetin, and 3,4-dihydroxyphenylpropionic acid. Mechanism analysis indicated that the disruption of tight structure, chemical bonds, and hemicellulose was crucial for the release of BPs from RPDF. This study provides valuable information on the potential application of SSF for the efficient release of BPs from RPDF, contributing to the utilization of RPDF as a functional food ingredient.

## 1. Introduction

*Rosa roxburghii* Tratt (RRT) belongs to the *Rosaceae* family and is primarily cultivated in the mountainous regions of southwestern China. This plant, locally known as ‘Cili’, is highly esteemed for its nutritional and medicinal properties. The fruit of RRT is abundant in various bioactive compounds, including polysaccharides, polyphenols, dietary fiber, and vitamins, making it a promising candidate for the development of functional food products [1]. In the processing industry of *R. roxburghii* fruit, a substantial amount of *R. roxburghii* fruit pomace (RRTP) is generated during juice processing, constituting nearly 50% of the whole fruit. However, RRTP is largely discarded as waste, leading to a significant loss of valuable resources [2]. RRTP is abundant in dietary fiber, with insoluble dietary fiber comprising approximately 70% of the RRTP content [3]. A recent study has revealed that *Rosa roxburghii* pomace insoluble dietary fiber (RPDF) demonstrates antioxidant and prebiotic activity, with bound polyphenols (BPs) playing a crucial role in contributing to the biological properties of RPDF [3]. Emerging studies have provided compelling evidence that the nutritional and biological properties of dietary fibers are greatly influenced by the presence of BPs [4,5].

BPs are often linked to biological macromolecules, particularly dietary fiber, through ester, ether, or glycoside bonds. Consequently, it is difficult to extract BPs directly with organic solvents, and common techniques for releasing BPs from natural sources include alkali hydrolysis and enzymatic hydrolysis [6,7]. Alkaline hydrolysis effectively breaks the ether and ester bonds between polyphenols and cell wall substances. While this method can extract BPs effectively, it may lead to the loss of BPs caused by harsh conditions and is also environmentally unfriendly. Enzymatic methods, on the other hand, offer mild conditions, avoiding the loss or degradation of BPs caused by extreme pH values or high temperatures. However, these methods have limitations such as low extraction rates and immaturity [8]. Therefore, there is an urgent need to explore suitable techniques for the efficient release of BPs from natural sources.

Solid-state fermentation (SSF) is a technique involving the cultivation of microorganisms on a solid substrate in a low-moisture environment with enough water to support the growth and metabolism of microorganisms [9]. During the fermentation process, microorganisms generate carbohydrate enzymes like ligninase, cellulase, or hemi-cellulase and utilize the carbohydrates present in the substrate, facilitating the gentle and efficient release of BPs [10]. SSF not only enables the release of substances that were difficult to extract from the substrate but also enhances the nutritional value of the substrate by producing additional nutrients [11,12,13]. *Aspergillus niger* is a filamentous fungus that possesses a complete cellulase system and can produce a large number of carbohydrate hydrolases, which have the ability to disrupt the intricate structure of dietary fibers and enhance the liberation of bioactive compounds [14]. *Aspergillus niger* has significant potential to be a candidate for biomass conversion and the production of bioactive compounds.

Therefore, the aim of the present study was to investigate the phytochemical profile, bioactivity, and release mechanism of BPs from RPDF by SSF with *Aspergillus niger*. The dynamic changes in the polyphenol composition of BPs and the structural characteristics of RPDF were examined. The findings of this study will provide valuable insights into the potential utilization of SSF for effectively releasing BPs from RPDF, thereby enhancing its application as a functional food ingredient.

## 2. Results and Discussion

### 2.1. Total Phenolic Content (TPC) of AT-RPDF-BP, HT-RPDF-BP, and FT-RPDF-BP

**Figure 1:**
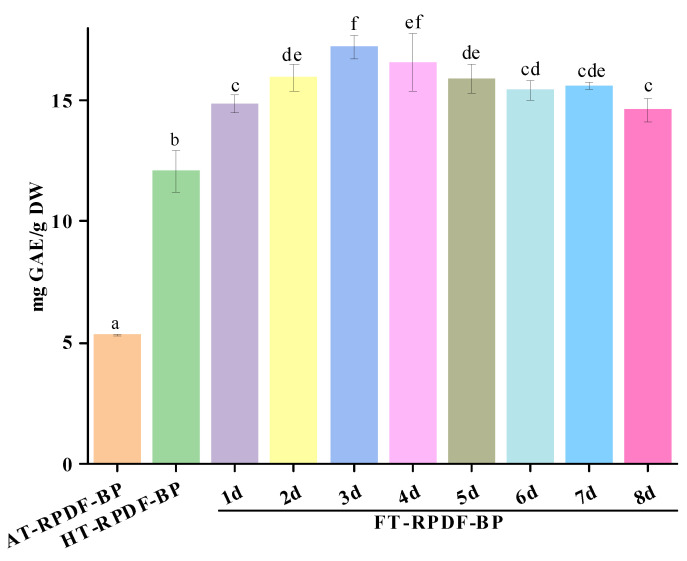
Total phenolic content (TPC) of bound polyphenols (BPs) released from RPDF using different methods at different time intervals. The BPs released from RPDF via alkaline hydrolysis, sterilization pretreatment, and solid-state fermentation with *Aspergillus niger* were labeled as AT-RPDF-BP, HT-RPDF-BP, and FT-RPDF-BP, respectively. Different letters indicate significant differences at a significance level of *p* < 0.05.

The BPs released by different extraction methods were designated as AT-RPDF-BP (alkaline hydrolysis), HT-RPDF-BP (sterilization pretreatment), and FT-RPDF-BP (solid-state fermentation with *Aspergillus niger*). As shown in Figure 1, the TPC of HT-RPDF-BP was 12.10 mg GAE/g DW, significantly higher than that of AT-RPDF-BP (5.33 mg GAE/g DW). Throughout the whole fermentation period, there was a continuous liberation of BPs, with the peak TPC content observed on the third day. FT-RPDF-BP on the third day (3d) displayed the highest TPC value (17.22 mg GAE/g DW), markedly exceeding AT-RPDF-BP and HT-RPDF-BP, respectively.

High-pressure sterilization as a pretreatment process was found to enhance the effective implementation of SSF by eliminating miscellaneous bacteria that could impede the growth of fermentation strains. Generally, the impact of sterilization on polyphenol release has been ignored. Dewanto et al. [15] reported that heat treatment at 115 °C for 25 min could elevate ferulic acid content and TPC in sweet corn by 550% and 54%, respectively. Cheng et al. [16] reported that heat treatment of wheat flour at 100 °C could boost the content of certain phenolic acids, such as ferulic acid, syringic acid, vanillic acid, and p-coumaric acid. In this study, we observed a significant increase in the TPC of BPs released from RPDF following sterilization (*p* < 0.05). The phenomenon may be attributed to the fact that sterilization disrupted the tight structure of the cell wall fiber network, facilitating the release of BPs.

The TPC of FT-RPDF-BP was higher than AT-RPDF-BP, aligning with previous studies [10,14]. The increase in TPC could be attributed to the effective breakdown of the RPDF structure by the growth of *Aspergillus niger* and its enzymes, leading to the continuous and efficient release of BPs [17]. However, the decline in TPC after the third day of fermentation might be due to the exhaustion of nutrients causing a stress response in fermentation fungi and the degradation or polymerization of phenolic substances [18,19].

### 2.2. Identification and Quantification of Bound Polyphenols

The composition and content of major polyphenols in AT-RPDF-BP, HT-RPDF-BP, and FT-RPDF-BP were analyzed. As displayed in Table 1, the polyphenolic composition of AT-RPDF-BP mainly consisted of gallic acid, gallocatechin, epigallocatechin, catechin, ellagic acid, and epicatechin, with small amounts of hydroxybenzoic acid, ferulic acid, and quercetin. These findings were consistent with a previous study on BPs extracted from RRTP [2]. After sterilization, HT-RPDF-BP exhibited the highest ellagic acid content, which was 9.29-times greater than that of AT-RPDF-BP. Conversely, the contents of gallic acid and epicatechin in AT-RPDF-BP were 3.31- and 3.58-times higher than in HT-RPDF-BP, respectively. Notably, gallic acid, *p*-hydroxybenzoic acid, ferulic acid, and quercetin were not detected in HT-RPDF-BP. The high content of ellagic acid in HT-RPDF-BP suggests its potential for extraction and preparation due to the highly heat-resistant characteristics of ellagic acid [20].

During solid-state fermentation, certain polyphenols like gallic acid, epigallocatechin, catechin, and ferulic acid were not detected, and epicatechin was only detected on the first day. The content of ellagic acid decreased from 26.37 to 4.23 mg/g during fermentation, which might be decomposed into other substances by enzymes. Hydroxybenzoic acid was detected on the third day, increasing to 9.97 mg/g by the seventh day, likely related to benzoic acid metabolism pathway and its association with phenyl acid metabolism pathways [14]. Quercetin was detected on the third day, steadily increasing to 5.09 mg/g by the seventh day. Additionally, 3,4-dihydroxy-phenylpropionic acid, absent in AT-RPDF-BP and HT-RPDF-BP, was identified in FT-RPDF-BP. Its content peaked at 1.94 mg/g on the second day, possibly due to the transformation of catechin or epicatechin by related enzymes [21]. Overall, the contents of epigallocatechin, hydroxybenzoic acid, quercetin, and 3,4-dihydroxy-phenylpropionic acid during fermentation were significantly higher than those in AT-RPDF-BP and HT-RPDF-BP.

### 2.3. Antioxidant Activity

**Figure 2:**
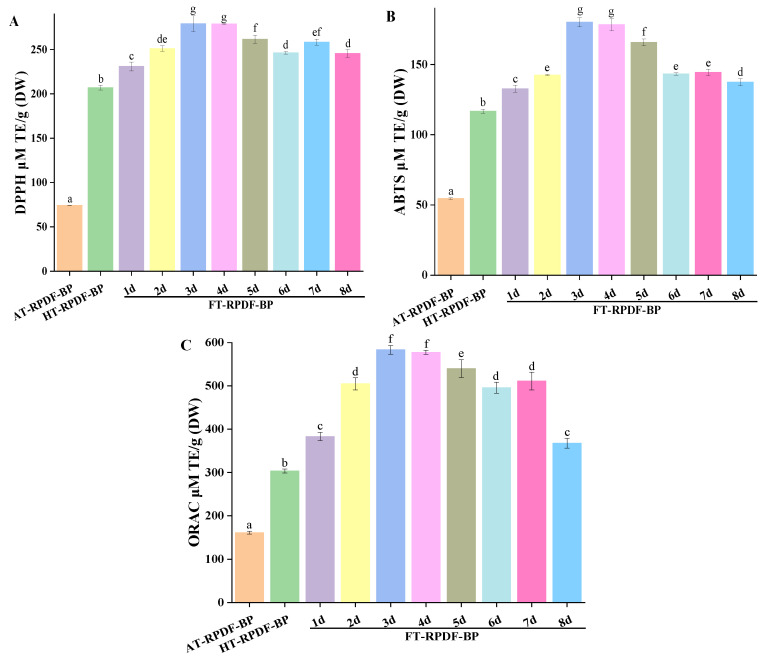
Antioxidant activity of bound polyphenols (BPs) released from RPDF using different methods at different time intervals: (**A**) DPPH radical scavenging activity; (**B**) ABTS radical scavenging activity; and (**C**) ORAC. The BPs released from RPDF via alkaline hydrolysis, sterilization pretreatment, and solid-state fermentation with *Aspergillus niger* were labeled as AT-RPDF-BP, HT-RPDF-BP, and FT-RPDF-BP, respectively. Different letters indicate significant differences at a significance level of *p* < 0.05.

DPPH, ABTS, and ORAC assays were conducted to evaluate the in vitro antioxidant activities of AT-RPDF-BP, HT-RPDF-BP, and FT-RPDF-BP. As shown in Figure 2, FT-RPDF-BP exhibited higher antioxidant activity than AT-RPDF-BP and HT-RPDF-BP. The antioxidant effects of HT-RPDF-BP were 207.10 (DPPH), 116.69 (ABTS), and 303.36 (ABTS) μmol TE/g DW, which were 2.79-, 2.14-, and 1.86-times higher than AT-RPDF-BP. Notably, FT-RPDF-BP (3d) exhibited the strongest antioxidant effect, with DPPH, ABTS, and ORAC values of 279.08, 180.07, and 583.48 μmol TE/g DW. During the solid-state fermentation process from 4 to 8 days, the antioxidant activity of FT-RPDF-BP decreased gradually, possibly due to the utilization or transformation of antioxidant substances. Saharan et al. studied the impacts of the fermentation process on cereal polyphenols and their antioxidant activity, revealing a positive correlation between the content of polyphenols and antioxidant activity [22]. In this study, Pearson’s correlation coefficient analysis showed that the TPC was positively correlated with the ORAC (r = 0.921, *p* < 0.01), DPPH free radical scavenging ability (r = 0.991, *p* < 0.01), and ABTS free radical scavenging ability (r = 0.957, *p* < 0.01).

### 2.4. Inhibitory Activities of α-Glucosidase

Type 2 diabetes mellitus is characterized by insufficient insulin production or ineffective utilization of insulin. Currently, numerous hypoglycemic agents are available to effectively regulate hyperglycemia. However, the clinical side effects associated with these agents, including gastrointestinal discomfort, diarrhea, and liver issues, should not be overlooked. Dietary polyphenols have been demonstrated to be used as effective supplements for controlling and preventing diabetes [23]. Inhibition of carbohydrate hydrolase activity, particularly α-glucosidase, is the most intuitive therapeutic approach.

Acarbose was employed as the positive control, and the experimental results were reported as semi-inhibitory concentration (IC_50_) values. As presented in Table 2, the IC_50_ values of AT-RPDF-BP, HT-RPDF-BP, FT-RPDF-BP (3d), and acarbose were 6.03, 4.16, 0.29, and 19.04 μg/mL, respectively. The α-glucosidase inhibitory activities of AT-RPDF-BP, HT-RPDF-BP, and FT-RPDF-BP (3d) were significantly superior to that of acarbose, with FT-RPDF-BP exhibiting the highest activity. Wang et al. [24] investigated the α-glucosidase inhibitory activity of methanol extract from guava leaves co-fermented by *Monascus anka* GIM 3.592 and Saccharomyces cerevisiae GIM 2.139 and found the IC_50_ value to be 0.09 mg/mL. Xie et al. [10] found that the IC_50_ value for α-glucosidase inhibition by BPs released through *Trichoderma viride* fermentation of rice bran insoluble dietary fiber was 7.36 μg/mL. Therefore, FT-RPDF-BP (3d) not only demonstrated superior α-glucosidase inhibitory activity compared to acarbose but also surpassed other polyphenol extracts released through SSF. These results suggest that FT-RPDF-BP (3d) has potential as a hypoglycemic candidate.

### 2.5. Microstructural Comparison of RPDF, HT-RPDF, and FT-RPDF

**Figure 3:**
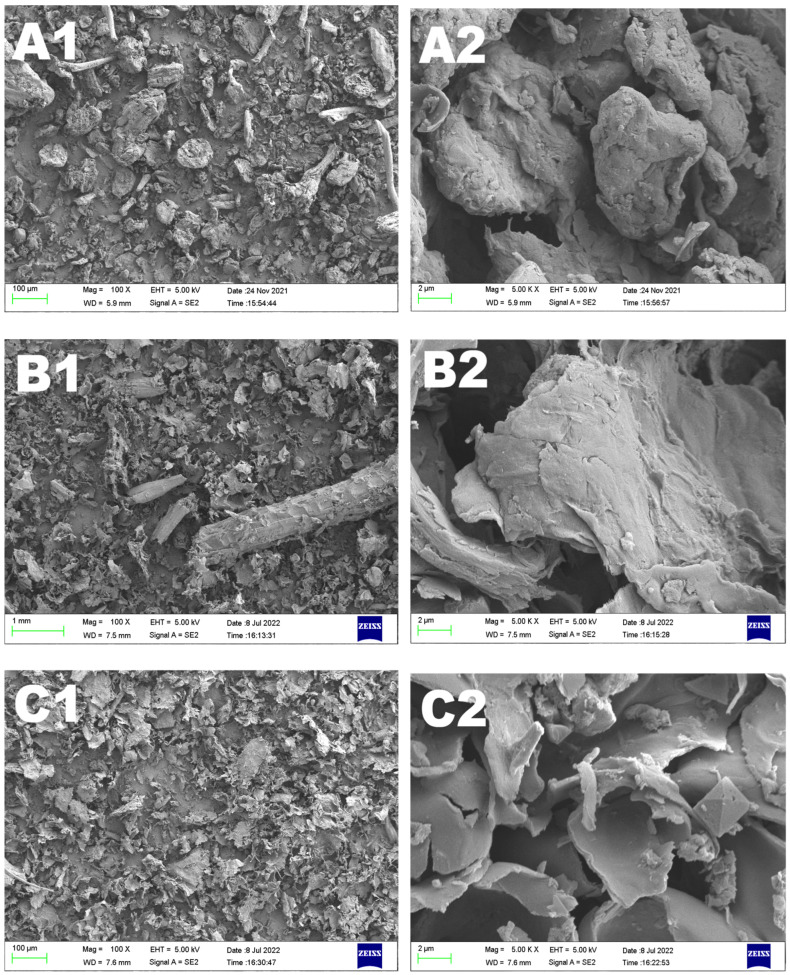
Scanning electron microscope (SEM) images of RPDF (**A1**,**A2**), HT-RPDF (**B1**,**B2**), and FT-RPDF (**C1**,**C2**). *Rosa roxburghii* pomace insoluble dietary fiber was labeled as RPDF. High-pressure sterilization-treated RPDF was labeled as HT-RPDF, and solid-state fermentation-treated RPDF was labeled as FT-RPDF-BP. Left images: 500× magnification; right images: 5000× magnification.

The microstructural differences among RPDF, HT-RPDF, and FT-RPDF are shown in Figure 3. Native RPDF exhibited a dense block-like structure, HT-RPDF appeared flaky and slightly curled with some lumps, while FT-RPDF displayed a sheet-like structure. Compared to the native RPDF, FT-RPDF had a thinner sheet structure. These observations indicated that the compact structure of RPDF was disrupted during sterilization and SSF, resulting in an increased contact area between the solvent and RPDF during the extraction process. This expanded contact area was conducive to the release of BPs from RPDF.

### 2.6. FT-IR Changes in RPDF, HT-RPDF, and FT-RPDF

**Figure 4:**
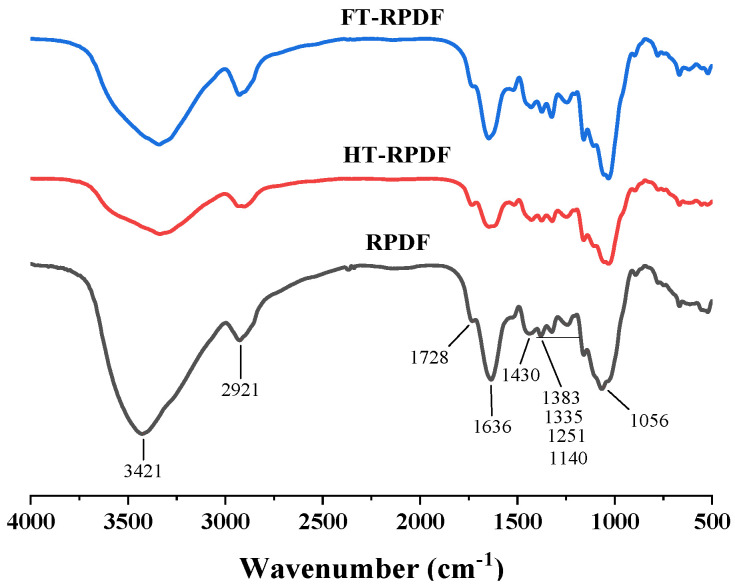
Fourier-transform infrared spectroscopy (FT-IR) spectra of RPDF, HT-RPDF, and FT-RPDF. *Rosa roxburghii* pomace insoluble dietary fiber was labeled as RPDF. High-pressure sterilization-treated RPDF was labeled as HT-RPDF, and solid-state fermentation-treated RPDF was labeled as FT-RPDF-BP.

The FT-IR spectra of RPDF, HT-RPDF, and FT-RPDF are illustrated in Figure 4. The characteristic peaks were identified based on the stretching vibration of specific groups or structures [25]. The strong absorption peak at 3421 cm^−1^ was the typical structure of cellulose and hemicellulose. The weak absorption peak at 2921 cm^−1^ was attributed to the stretching vibration of methyl or methylene groups, which were typical structures of cellulose [26]. The absorption peaks at around 1728 cm^−1^ and 1636 cm^−1^ were assigned to the stretching vibration of the C=O bond in the acetyl carboxyl group of hemicellulose and the methyl esterification or free carboxyl group of pectin, respectively [27]. The absorption peak at 1430 cm^−1^ was attributed to aliphatic or aromatic C-H vibration, which was the typical structure of lignin. Another set of absorption peaks, primarily from cellulose and hemicellulose, was located around 1100–1300 cm^−1^ with the contraction vibration of the C-O group. The broad peaks at approximately 1056 cm^−1^ were attributed to the stretching vibration of the C-O-C bond, suggesting the existence of a pyranose ring, particularly evident in arabinoxylan or xylan [28]. These characteristic peaks revealed that RPDF, HT-RPDF, and FT-RPDF exhibited typical dietary fiber structures, containing cellulose, hemicellulose, lignin, and pectin. While RPDF, HT-RPDF, and FT-RPDF displayed similar spectral profiles, there were variations in the absorption intensities of their characteristic peaks. The fluctuation intensity of HT-RPDF and FT-RPDF was weaker than that of RPDF at 3421 cm^−1^, suggesting that the intermolecular hydrogen bonds in cellulose and hemicellulose were disrupted after sterilization and SSF. BPs were often closely connected with dietary fiber through the interaction of ester bonds, ether bonds, hydrophobic bonds, and hydrogen bonds [29]. The destruction of these chemical bonds led to the release of BPs. Therefore, after sterilization and SSF, the functional groups or cell walls in RPDF were destroyed, affecting the connection between BPs and dietary fiber and consequently leading to the release of BPs from RPDF.

### 2.7. Thermal Properties of RPDF, HT-RPDF, and FT-RPDF

**Figure 5:**
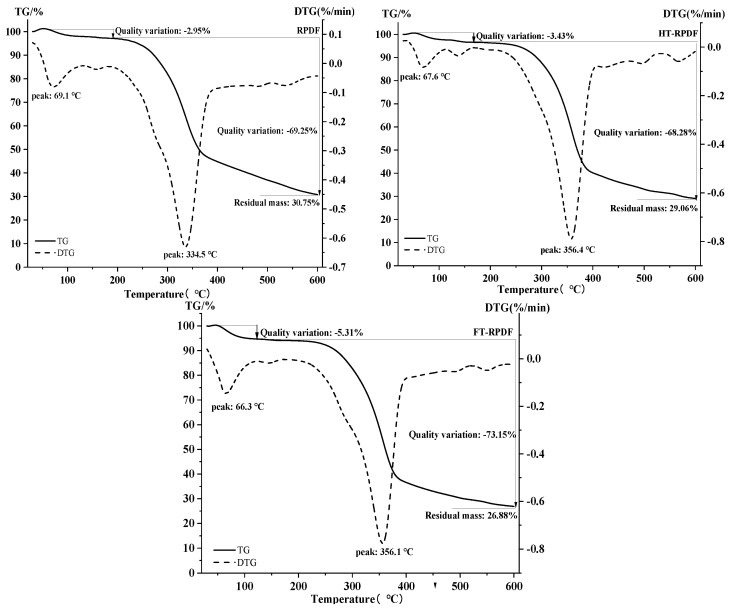
Thermogravimetric (TG) curves of RPDF, HT-RPDF, and FT-RPDF. *Rosa roxburghii* pomace insoluble dietary fiber was labeled as RPDF. High-pressure sterilization-treated RPDF was labeled as HT-RPDF, and solid-state fermentation-treated RPDF was labeled as FT-RPDF-BP.

The TG and DTG curves of RPDF, HT-RPDF, and FT-RPDF are shown in Figure 5. The weight loss of RPDF, HT-RPDF, and FT-RPDF was divided into two stages. In the first stage (30~200 °C), RPDF, HT-RPDF, and FT-RPDF exhibited distinct endothermic peaks at 69.1, 67.6, and 66.3 °C, respectively. This initial stage saw a mass reduction of about 3~6%, indicating the loss of free water from the dietary fiber as the temperature increased. Additionally, a weak absorption peak between 100 and 200 °C was observed, which might be the loss of bound water [30]. In the second stage (200~600 °C), RPDF, HT-RPDF, and FT-RPDF displayed obvious endothermic peaks in the DTG curve, accompanied by a noticeable downward trend in the TG curve. This stage was attributed to the degradation of carbohydrates, including cellulose, hemicellulose, and pectin [31]. Previous studies have indicated that the carbonization process of hemicellulose, cellulose, and lignin was exothermic, with the pyrolysis peak of hemicellulose at 270–310 °C, cellulose at 320–370 °C, and lignin at above 400 °C [32]. The endothermic peaks of RPDF, HT-RPDF, and FT-RPDF were obviously different at 200~600 °C, indicating a reduction in hemicellulose content after sterilization and SSF. This disruption of the interaction between hemicellulose, cellulose, and lignin led to the release of BPs in RPDF [33]. Additionally, the residual mass of HT-RPDF and FT-RPDF was lower than that of RPDF, consistent with the trend observed in crystallinity. Therefore, sterilization and SSF could affect the components of RPDF, disrupt the connection between BPs and dietary fiber, and facilitate the release of BPs from RPDF.

## 3. Materials and Methods

### 3.1. Experimental Materials

Dried *R. roxburghii* fruit pomace was kindly provided by Guizhou Xinyang Agricultural Science and Technology Development Co., Ltd. (Guizhou, China). RPDF was extracted from *R. roxburghii* fruit pomace following our previously reported protocol [3]. *Aspergillus niger* (GDMCC 3.576) was purchased from Guangdong Microbial Culture Collection Center. Gallic acid, gallocatechin, epigallcatechin, catechin, hydroxybenzoic acid, epicatechin, ellagic acid, ferulic acid, quercetin, and 3,4-dihydroxyphenylpropionic acid were purchased from Solarbio Co., Ltd. (Shanghai, China). Unless stated otherwise, all other chemical reagents were of analytical grade or higher quality.

### 3.2. Sterilization Pretreatment of RPDF

The fermentation substrate was prepared by mixing 10 g of RPDF powder with 90 mL of distilled water in a conical flask. The substrate was then sterilized at 121 °C for 15 min. After the sterilization treatment, the sample was cooled on a clean bench for inoculation and then stored in a refrigerator at −80 °C. This sample was labeled as HT-RPDF for subsequent analysis.

### 3.3. Solid-State Fermentation of RPDF

First, *Aspergillus niger* was incubated on potato dextrose agar media at 27 °C for 3 days to activate the strain. After activation, 10 mL of sterile distilled water was added to scrape spores, and sterile cotton was used to filter hyphae to collect spore suspension. The spore suspension was then adjusted to a concentration of 2 × 10^7^ spores/mL for inoculation. Subsequently, 0.5 mL of the diluted spore suspension was added to a sterilized conical flask containing 1 g of the HT-RPDF sample. The fermentation medium was then incubated at 27 °C for 8 days, with samples taken every 24 h. The fermentation HT-RPDF sample, designated as FT-RPDF, was stored at −80 °C for subsequent investigations at each time point.

### 3.4. Extraction of Bound Polyphenols

Three BPs released by different extraction methods were designated as AT-RPDF-BP (alkaline hydrolysis), HT-RPDF-BP (sterilization pretreatment), and FT-RPDF-BP (solid-state fermentation with *Aspergillus niger*).

The extraction procedures of HT-RPDF-BP and FT-RPDF-BP from HT-RPDF and FT-RPDF were carried out following our previously reported protocol [2]. In detail, each dietary fiber sample was mixed with 70% ethanol (1:15, *v*/*v*) and shaken in a shaker for 1 h. The resultant supernatant was collected by centrifugation at 4500× *g* for 5 min. The residue was repeated 6 times under the same conditions. All the collected supernatants were concentrated, lyophilized, and re-dissolved in pure methanol for further analysis.

The extraction of AT-RPDF-BP from RPDF was conducted by the alkaline hydrolysis method [4,34]. In brief, 1 g of RPDF was mixed with 10 mL of 4 mol/L NaOH solution under an oxygen-free atmosphere. The mixed solution was stirred slowly for 4 h at room temperature in the absence of light. Subsequently, the pH of the mixture was acidified to 2.0 using 6 mol/L HCl, and it underwent six extractions with ethyl acetate (1:1, *v*/*v*). The organic fraction was obtained by centrifugation at 4500× *g* for 5 min, concentrated, and then freeze-dried.

### 3.5. Determination of Total Phenolic Content

The total phenolic content (TPC) in AT-RPDF-BP, HT-RPDF-BP, and FT-RPDF-BP was determined using the Folin–Ciocalteu reagent method [3]. A standard curve was generated using gallic acid at concentrations ranging from 50 to 500 μg/mL. The result was expressed as milligrams of gallic acid equivalent (GAE) per gram of dry weight (DW) of the extract (mg GAE/g DW).

### 3.6. Quantification of Individual Phenolic Compound Contents

The contents of individual phenolic compounds in AT-RPDF-BP, HT-RPDF-BP, and FT-RPDF-BP were determined following a previously established method [2]. The composition and content of phenolic compounds were quantified using an Agilent 1260 system equipped with a ZORBAX SB-C18 (4.6 × 250 mm, 5 μm) column and a diode array detector (DAD). The mobile phases comprised 0.1% formic acid in an aqueous solution and acetonitrile, with a flow rate of 0.8 mL/min. The injection volume and column temperature were set at 50 μL and 30 °C, respectively. Standards including gallic acid, ellagic acid, urolithin A, 3,4-dihydroxyphenylpropionic acid, and epicatechin were utilized at concentrations ranging from 10 to 200 μg/mL. Results were presented as micrograms per gram based on DW (μg/g DW).

### 3.7. Determination of Antioxidant Activity

#### 3.7.1. DPPH Radical Scavenging Activity

The DPPH scavenging activity of the samples was assessed following a previously described protocol [3]. Briefly, 100 μL of different concentrations of sample solutions or Trolox (6.25–100 μM) were mixed with 100 μL of DPPH solution (0.2 mM) in 96-well plates. The mixtures were then incubated in darkness at room temperature for 30 min. The reaction of the distilled water and DPPH solution was set as a control. The absorbance of the reaction mixture was measured at 517 nm. A standard curve was constructed using Trolox, and the results were expressed as μM Trolox equivalent (TE) per gram of DW.

#### 3.7.2. ABTS Radical Scavenging Activity

The ABTS·^+^ scavenging activity of the samples was determined by a previously described method with slight modifications [3]. Briefly, 50 μL of different concentrations of sample solutions or Trolox (6.25–100 μM) were mixed with 150 μL of ABTS·^+^ working solution (OD value: 0.7 at 734 nm) in 96-well plates. After incubating for 6 min at room temperature, the absorbance at 734 nm was measured using a microplate reader (Biotek, Winooski, VT, USA). Results were reported as μM Trolox equivalent (TE) per gram of DW.

#### 3.7.3. Oxygen Radical Absorbance Capacity (ORAC)

The ORAC assay was conducted following a previously reported protocol with some modifications [3]. Briefly, 20 μL of sample solution and 200 μL of fluorescein sodium salt solution (95.6 nM) were added and mixed automatically in 96-well plates. The mixture was then incubated for 15 min at 37 °C. Upon initiating the reaction by adding 20 μL of AAPH (119.4 mM), the fluorescence intensity of the system was recorded every 3 min for 35 cycles at a λ_excitation_ of 485 nm and λ_emission_ of 538 nm. A standard curve was constructed using different concentrations of Trolox solution (6.25–100 μM), and the results were presented as μM Trolox equivalent (TE) per gram of DW (μM TE/g DW).

### 3.8. Determination of α-Glucosidase Inhibitory Activity

The α-glucosidase inhibitory activity of the samples was assessed following a previously described method [3]. Briefly, 50 μL of acarbose or BPs samples at varying concentrations and 50 μL of α-glucosidase (0.5 U/mL) were incubated in 96-well plates for 10 min at room temperature. Following pre-incubation, 50 μL of *p*-nitrophenyl-α-D-glucopyranoside (pNPG, 5 mM) was added to initiate the reaction. After a 15 min incubation, 100 μL of Na_2_CO_3_ (0.2 M) was added to terminate the reaction. The absorbance of the mixtures was then measured at 405 nm. Acarbose served as the positive control, while the reaction without α-glucosidase served as the blank control. The inhibition rate was calculated using the Formula (1):

Inhibitory rate (%) = (1 − (A_S_ − A_B_)/(A − A_C_)) × 100 (1)

where A_S_ and A represent the absorbance values of the reaction system with and without samples, and A_B_ and A_C_ present the absorbance values of the blank and control samples, respectively.

### 3.9. Structural Analysis

To investigate the mechanism of releasing BPs through different treatments, dietary fiber samples including native RPDF (RPDF), after RPDF sterilization (HT-RPDF), and RPDF after solid-state fermentation (FT-RPDF-BP) were analyzed.

#### 3.9.1. Surface Morphology

The surface morphology of RPDF, HT-RPDF, and FT-RPDF was observed using a scanning electron microscope (Zeiss, Jena, Germany). Images of the tested samples were captured at magnifications of 100× and 5000×, respectively.

#### 3.9.2. Fourier-Transform Infrared Spectroscopy (FT-IR)

The FT-IR spectral characteristics of RPDF, HT-RPDF, and FT-RPDF were analyzed using an FT-IR microscope (Thermo Fisher Scientific, Waltham, MA, USA). Briefly, 3 mg of each sample was thoroughly mixed with 150 mg of KBr power in an agate mortar, ground, and then pressed into about 1 mm thick pellets. The resulting pellets were scanned in the range of 400–4000 cm^−1^ using Nicolet IS50-Nicolet Continuum (Thermo Scientific, Waltham, MA, USA).

#### 3.9.3. Thermos Gravimetric (TG)

The thermal properties of RPDF, HT-RPDF, and FT-RPDF were examined using a TGA simultaneous thermal analyzer (PE STA 8000, PerkinElmer, Shelton, CT, USA). TG analysis was performed according to a previously described method. RPDF, HT-RPDF, and FT-RPDF (5–10 mg) were placed in an alumina crucible, with an empty crucible used as a reference. The heating temperature ranged from 30 to 600 °C at a rate of 10 °C/min under a N2 atmosphere.

### 3.10. Statistical Analysis

The data are presented as the means ± SD with three replicates. Statistical significance between groups was determined using one-way analysis of variance (ANOVA) with SPSS software 25.0 (SPSS Inc., Chicago, IL, USA), where differences were considered as significant at *p* < 0.05.

## 4. Conclusions

In conclusion, this study demonstrates that solid-state fermentation with *Aspergillus niger* can efficiently and environmentally facilitate the release of bound polyphenols (BPs) from *Rosa roxburghii* fruit pomace insoluble dietary fiber (RPDF). The BPs released through solid-state fermentation exhibited superior antioxidant and α-glucosidase inhibitory activities compared to that released through alkaline hydrolysis. Structural analysis revealed that sterilization and solid-state fermentation disrupted the tight structure and certain chemical bonds within RPDF, leading to the breakdown of the connection between BPs and RPDF. Additionally, the reduction in hemicellulose content affected its interaction with other fiber components. These structural changes facilitated the release of BPs from RPDF. Overall, this study provides valuable insights into the potential application of solid-state fermentation for the efficient release of BPs from RPDF, thereby contributing to the utilization of RPDF as a functional food ingredient.

## Figures and Tables

**Table 1:** Compositions and contents of bound polyphenols (BPs) released from RPDF using different methods ^a^.

Composition	AT-RPDF-BP	HT-RPDF-BP	FT-RPDF-BP							
			1d	2d	3d	4d	5d	6d	7d	8d
Gallic acid	1.19	0.36 ± 0.02	ND	ND	ND	ND	ND	ND	ND	ND
Gallocatechin	0.88 ± 0.01	ND	ND	ND	ND	ND	ND	ND	ND	ND
Epigallcatechin	0.24 ± 0.02 ^a^	0.40 ± 0.01 ^a^	1.06 ± 0.08 ^b^	1.92 ± 0.14 ^e^	1.56 ± 0.18 ^c^	1.62 ± 0.05 ^cd^	2.04 ± 0.05 ^e^	1.81 ± 0.13 ^de^	ND	ND
Catechin	0.18 ± 0.02	0.23	ND	ND	ND	ND	ND	ND	ND	ND
Hydroxybenzoic acid	1.18 ± 0.02 ^a^	ND	ND	ND	4.10 ± 0.09 ^b^	5.53 ± 0.03 ^c^	6.95 ± 0.02 ^d^	8.05 ± 0.01 ^e^	9.97 ± 0.17 ^g^	8.58 ± 0.04 ^f^
Epicatechin	1.36 ± 0.07 ^b^	0.38 ± 0.01 ^a^	0.37 ± 0.01 ^a^	ND	ND	ND	ND	ND	ND	ND
Ellagic acid	2.84 ± 0.08 ^a^	26.37 ± 0.02 ^h^	14.98 ± 0.02 ^g^	7.87 ± 0.32 ^f^	6.63 ± 0.22 ^e^	6.42 ± 0.10 ^e^	5.60 ± 0.02 ^d^	4.27 ± 0.04 ^b^	4.72 ± 0.01 ^c^	4.23 ± 0.04 ^b^
Ferulic acid	0.07 ± 0.01	ND	ND	ND	ND	ND	ND	ND	ND	ND
Quercetin	0.07 ^a^	ND	ND	ND	0.23 ± 0.01 ^b^	2.29 ± 0.02 ^c^	3.45 ± 0.03 ^d^	4.09 ± 0.02 ^e^	5.09 ± 0.03 ^g^	4.59 ± 0.01 ^f^
3,4-Dihydroxy phenylpropionic acid	ND	ND	1.41 ± 0.04 ^c^	1.94 ± 0.14 ^d^	1.33 ± 0.07 ^bc^	1.27 ± 0.04 ^b^	1.36 ± 0.04 ^bc^	0.91 ± 0.04 ^a^	0.89 ± 0.04 ^a^	0.80 ± 0.02 ^a^

^a^ The BPs released from RPDF via alkaline hydrolysis, sterilization pretreatment, and solid-state fermentation with *Aspergillus niger* were labeled as AT-RPDF-BP, HT-RPDF-BP, and FT-RPDF-BP, respectively. Values with different letters in the same line are significantly different at *p* < 0.05. ND: not detected. All data units in the table are μg/g DW.

**Table 2:** Inhibitory activity of α-glucosidase of bound polyphenols released from RPDF by alkaline hydrolysis, high-pressure sterilization, and solid-state fermentation ^a^.

Sample	IC50 (μg/mL)
AT-RPDF-BP	6.03 ± 0.04 ^d^
HT-RPDF-BP	4.16 ± 0.09 ^c^
FT-RPDF-BP	0.29 ± 0.01 ^b^
Acarbose	19.04 ± 0.97 ^e^

^a^ The BPs released from RPDF via alkaline hydrolysis, sterilization pretreatment, and solid-state fermentation with *Aspergillus niger* were labeled as AT-RPDF-BP, HT-RPDF-BP, and FT-RPDF-BP, respectively. Acarbose was used as a positive control. Values with different letters are significantly different at *p* < 0.05.

## Data Availability

Data are contained in the article.
